# Transcriptome Analysis of the Effects of Compound Microecological Preparation on Chickens Challenged with Newcastle Disease Virus

**DOI:** 10.3390/ijms27135771

**Published:** 2026-06-26

**Authors:** Xinxin Qiu, Zhencang Zhang, Wenhui Wang, Yanqing Jia

**Affiliations:** 1 The Youth Innovation Team of Shaanxi Universities, Shaanxi Engineering Research Center of Animal Disease Prevention and Control, Department of Animal Engineering, Shaanxi A&F Technology University, Yangling 712100, China; ylzyqiuxinxin@126.com (X.Q.); z13572436889@126.com (Z.Z.); 2 College of Veterinary Medicine, Gansu Agricultural University, Lanzhou 730070, China; wwh777@126.com

**Keywords:** compound microecological preparation, NDV, intestine, immunity

## Abstract

Newcastle disease (ND), a highly contagious poultry disease caused by NDV, primarily triggers gastrointestinal lesions. Microecological preparations, novel biological additives for restoring intestinal microbiota diversity, improve nutrient absorption, reinforce intestinal barrier function, and modulate host immune responses. This study investigated the effects of a compound microecological preparation on intestinal pathogenicity in chickens infected with genotype VII Newcastle disease virus (NDV). SPF chickens were allocated to four dietary groups with or without a compound microecological preparation, followed by NDV challenge in two groups. Survival, intestinal morphology, and transcriptomic responses were assessed. The results showed that chickens fed with the compound microecological preparation exhibited improved intestinal development. Following NDV infection, these chickens displayed milder cecal lesions without obvious hemorrhage and a higher survival rate. Furthermore, differential gene transcription analysis revealed that supplementation with the compound microecological preparation regulated the expression of genes associated with metabolic processes, biological regulation, immune response, and growth and development pathways, consistent with the clinical findings. In conclusion, we demonstrated that the compound microecological preparation promotes intestinal development in chickens, delays disease progression following NDV infection, and alleviates pathological damage. Transcriptomic analysis further indicated that the preparation enhances intestinal mucosal immunity by stimulating IgA production and strengthening the immune response against genotype VII NDV. These findings provide a scientific basis for the application of compound microecological preparations in regulating the intestinal immune system and in the prevention of Newcastle disease.

## 1. Introduction

Newcastle disease (ND), a highly contagious infectious disease of poultry caused by the ND virus (NDV), is characterized by damage to the digestive tract and central nervous system. Studies have shown that the main pathological changes in chickens infected with genotype VII NDV include splenic swelling and congestion accompanied by severe hemorrhagic necrosis and fibrous inflammation, as well as rectal swelling and hemorrhage and hemorrhagic inflammation of the mucosa. In China, sustained implementation of comprehensive immunization and prevention strategies against ND has effectively brought the epidemic under control [1]. However, viral contamination persists in wild birds, waterfowl populations, and the environment, and ND still exhibits endemic characteristics in some regions. Most cases occurring in vaccinated chicken flocks are mild and sporadic, which may be attributed to immune dysfunction. Atypical ND and the phenomenon of immune carrier status have been recognized for a long time. With the ban on antibiotics in poultry farming and the increasing complexity of poultry diseases, considerable challenges have arisen in the clinical prevention and control of avian diseases [2]. Therefore, the development of safe, effective, and environmentally sustainable alternatives to antibiotics is crucial.

Microecological agents, a novel class of biological additives designed to rebuild and optimize the diversity of animal gut microbiota, may promote nutrient absorption, enhance intestinal barrier function, and modulate the immune response of the host. Probiotics, as green microecological preparations, have been applied to improve intestinal health and prevent and control diseases and are widely used in the poultry industry, particularly as immunopotentiators. For example, Gu et al. [3] investigated the immunomodulatory effects of *Bacillus cereus* PAS38 (PB) on the spleen of broiler chickens. By comparing the relative weight of the spleen and histological sections, they found that PB significantly promoted the development of immune organs, and genes associated with inflammatory responses were notably downregulated. Onmei et al. [4] investigated the effects of two *Lactobacillus reuteri* PIA16 strains, isolated separately from the cecum and jejunum of native chickens in Assam, administered either alone or in combination with probiotics, on NDV immunity. They found that both treatments stimulated the humoral and cellular immune responses of the host and significantly increased NDV-specific antibody levels.

However, most existing studies have focused on single-strain probiotics or simple two-strain combinations, while compound microecological preparations integrating multiple beneficial bacteria and plant-derived immunomodulators often exhibit more comprehensive and synergistic protective effects. The compound microecological preparation used in this study was independently developed and prepared in our laboratory. Its core active components include *Bacillus subtilis*, *Bacillus licheniformis*, *Enterococcus faecium*, *Clostridium butyricum*, *Lactic acid bacteria*, and *Astragalus polysaccharides*. Among them, *Bacillus subtilis* and *Bacillus licheniformis* are spore-forming bacteria with strong gastrointestinal tolerance, which can produce various digestive enzymes and antibacterial substances to inhibit pathogenic colonization. *Enterococcus faecium* and *lactic acid bacteria* are dominant commensal bacteria that rapidly colonize the intestinal mucosa to regulate microbial balance and stimulate mucosal immunity. *Clostridium butyricum* produces butyrate, the main energy source for intestinal epithelial cells, to maintain mucosal barrier integrity and exert anti-inflammatory effects. Astragalus polysaccharides, as a natural immunomodulator, can enhance both innate and adaptive immune responses. This multi-component formula exerts complementary and synergistic effects: Bacillus strains improve the intestinal microenvironment to facilitate the colonization of lactic acid bacteria and Enterococcus faecium, while Astragalus polysaccharides further amplify the immunomodulatory effects of probiotics. Nevertheless, the molecular mechanisms by which this specific compound preparation protects against NDV-induced intestinal injury, particularly at the transcriptomic level, remain poorly understood.

This study aimed to use transcriptomic sequencing to investigate the effects of a compound microecological preparation on pathogenicity in chickens infected with genotype VII NDV, focusing on post-infection intestinal injury and regulation of intestinal immunity, thereby providing a scientific basis for its application in the prevention and control of ND.

## 2. Results

### 2.1. Survival Analysis and Histological Lesions

**Figure 1:**
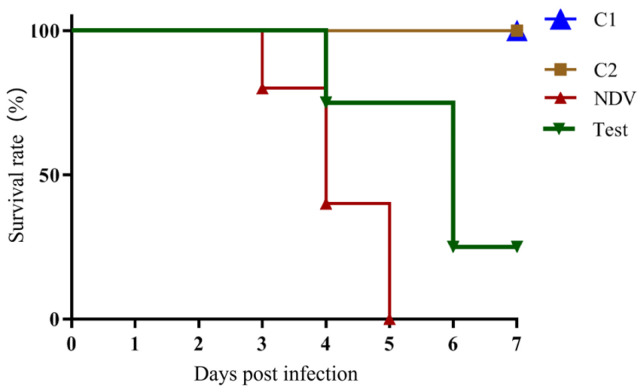
Survival curve analysis.

**Figure 2:**
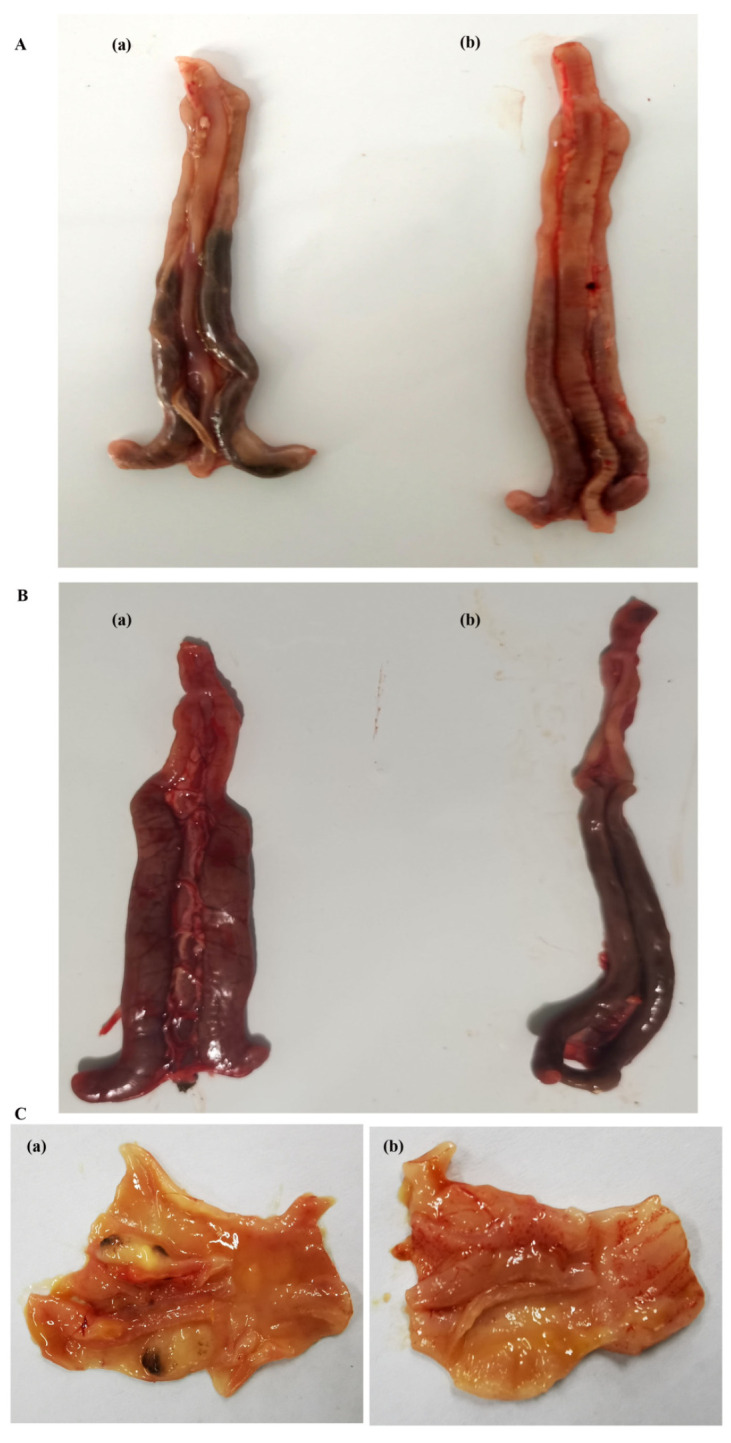
Comparative observation of pathological changes. (**A**) Comparison of the cecum between control groups C1 (**a**) and C2 (**b**). (**B**) Comparison of cecal lesions induced by NDV infection between the NDV (**a**) and Test groups (**b**). (**C**) Comparison of caecal tonsil lesions in response to NDV infection between the NDV (**a**) and Test groups (**b**). NDV, Newcastle disease virus.

Clinical observations showed that chickens in control groups C1 and C2 exhibited no obvious clinical signs. Mortality in the NDV group began on day 3 post-infection and continued thereafter, whereas mortality in the Test group was first observed on day 4 (Figure 1). Necropsies were performed on dead chickens from the control and infected groups. Chickens fed the compound microecological preparation exhibited improved gastrointestinal development. Following NDV infection, the Test group displayed milder cecal lesions with no obvious hemorrhage (Figure 2). Scoring and recording of clinical signs and pathological lesions were conducted for all chickens strictly following the standardized scoring system established in prior studies [1]. Complete individual scoring records are provided in the Supplementary Materials (Table S1). These results indicate that the compound microecological preparation exerts specific effects on promoting intestinal development and reducing NDV pathogenicity in chickens.

### 2.2. mRNA Differential Analysis

**Figure 3:**
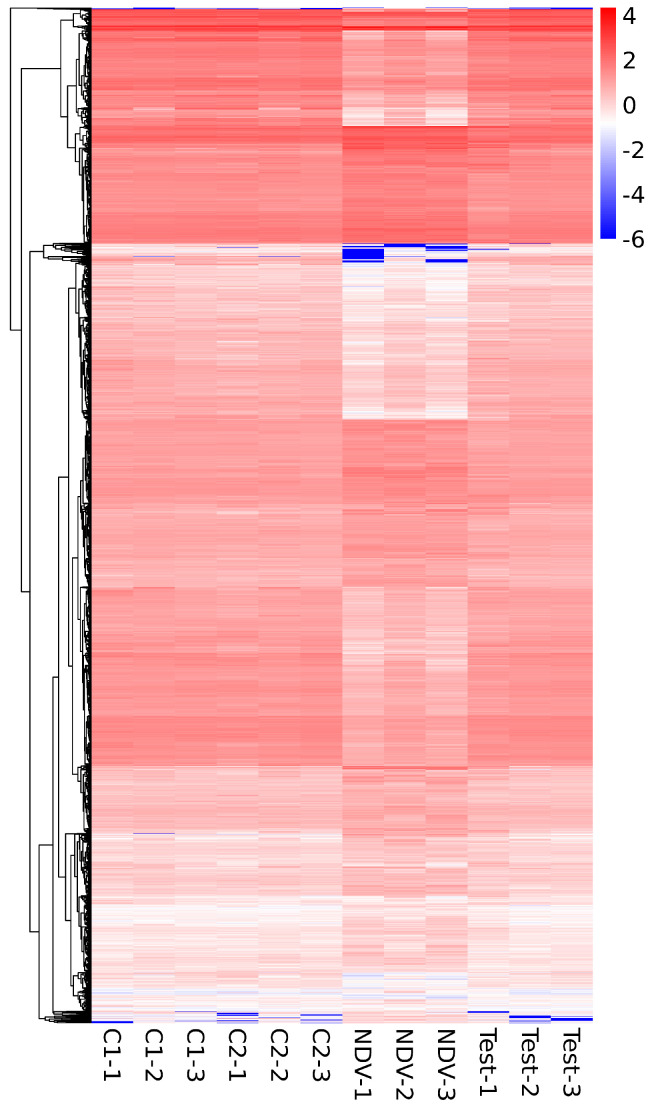
Cluster analysis of all differentially expressed genes.

For samples with biological replicates, the DESeq2 package was applied for differential expression analysis across sample groups to screen differentially expressed genes (DEGs) between two distinct biological treatments. During the detection of differentially expressed genes, the screening criteria were set as Log_2_ Fold Change ≥ 1 and *p*-value < 0.05. Cluster analysis was conducted on all screened differentially expressed genes, as shown in Figure 3.

**Figure 4:**
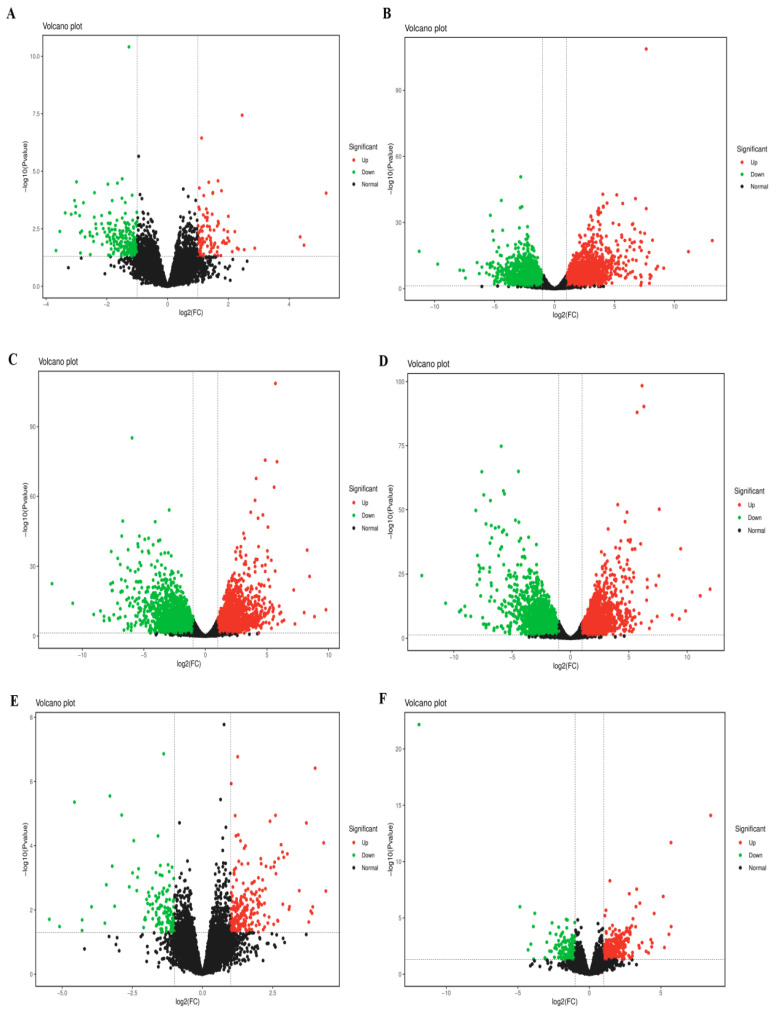
Volcano plots of differentially expressed genes. (**A**) C1_vs_C2; (**B**) NDV_vs_Test; (**C**) C1_vs_NDV; (**D**) C2_vs_NDV; (**E**) C1_vs_Test; (**F**) C2_vs_Test. Genes with significant differences are represented by red (upregulation) and green (downregulation) dots, and genes with no significant difference are represented by black dots. The abscissa represents gene expression fold changes across different samples, while the ordinate represents genes showing statistically significant differences in expression.

Volcano plots were used to intuitively display the differentially expressed genes (DEGs) between two groups of samples (Figure 4). The results showed that 329 DEGs were identified between control groups C1 and C2, including 104 upregulated and 225 downregulated genes; 5276 DEGs were identified between the C1 and NDV groups, with 2673 upregulated and 2603 downregulated genes; 356 DEGs were detected between the C1 and Test groups, including 214 upregulated and 132 downregulated genes; 5017 DEGs were identified between the C2 and NDV groups, of which 2588 were upregulated and 2429 were downregulated; 444 DEGs were observed between the C2 and Test groups, with 305 upregulated and 139 downregulated genes; 5080 DEGs were identified between the NDV and Test groups, including 2488 upregulated and 2592 downregulated genes (Table 1).

### 2.3. Verification of DEGs

**Figure 5:**
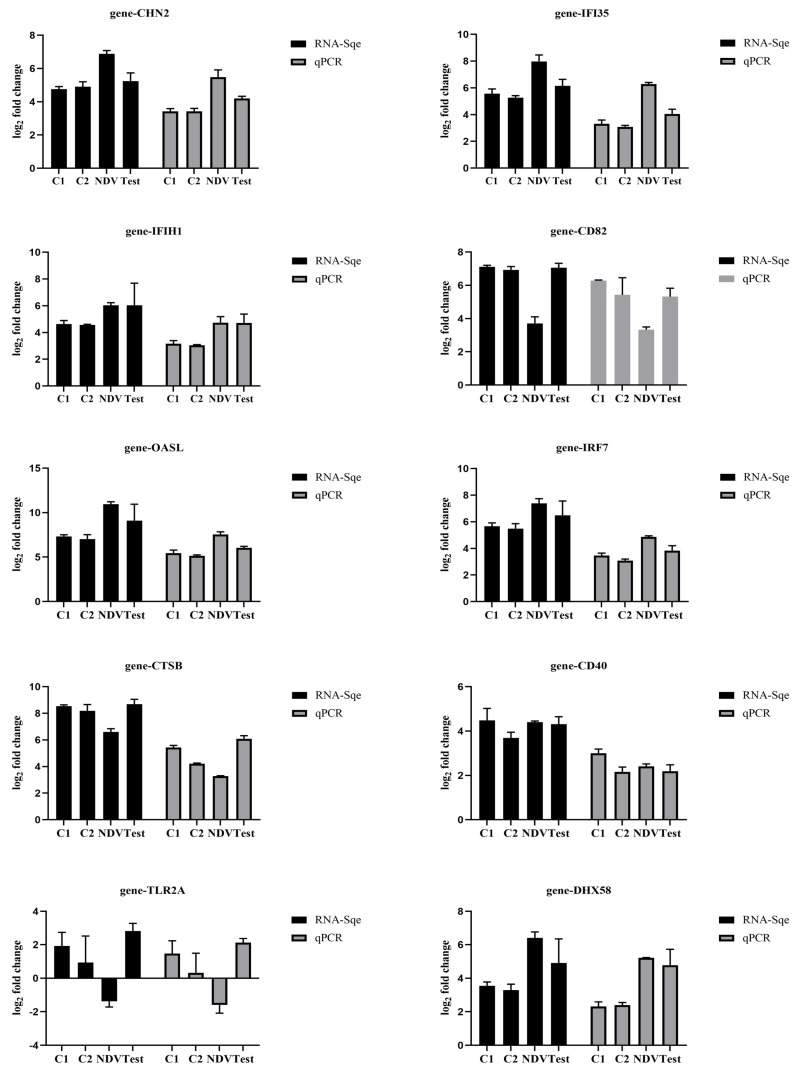
Validation of differentially expressed genes from transcriptome sequencing using qPCR.

With |Log_2_ Fold Change| > 1 as the screening criterion for DEGs, 10 DEGs were randomly selected and verified using qPCR. The qPCR validation results were consistent with those of transcriptome sequencing. The upregulated DEGs included *CHN2*, *DHX58*, *IFI35*, *IFIH1*, *CD82*, *CD40*, *OASL*, and *CTSB*. *TLR2A* was downregulated under NDV infection but upregulated in the other groups. These validation results indicated that the sequencing data were reliable and could be used for subsequent data analysis (Figure 5). The primers used for gene verification in this study are listed in Table 2.

### 2.4. Bioinformatics Analysis of DEGs

Functional annotation of DEGs was performed using database analysis, and statistics on the number of annotated genes in each DEG set are presented in Table 3.

**Figure 6:**
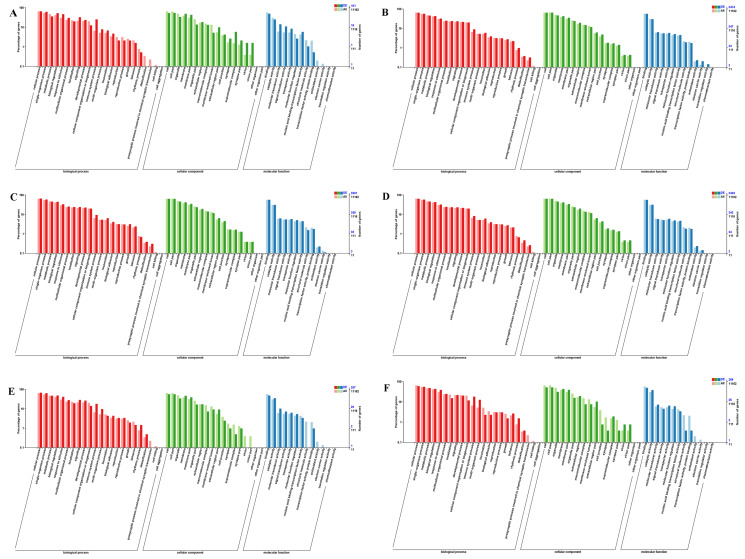
Gene Ontology (GO) analysis of differentially expressed genes across the three comparisons. (**A**) C1_vs_C2; (**B**) NDV_vs_Test; (**C**) C1_vs_NDV; (**D**) C2_vs_NDV; (**E**) C1_vs_Test; (**F**) C2_vs_Test.

Gene Ontology (GO) analysis was performed on DEGs, revealing distinct functional patterns among the comparison groups (Figure 6). The results showed that relative to those in the C2 group, DEGs in the C1 group were mainly enriched in the immune system, biological regulation, response to stimulus, signal transduction, growth and development, cell killing, and viral particle, and related functional categories (Figure 6A). Compared with those in the NDV group, DEGs in the C1 group were mainly enriched in growth and development, biological regulation, cell junction, catalytic activity, and related functional categories (Figure 6B), whereas comparison with the Test group highlighted enrichment in the immune system, detoxification, extracellular region components, multi-organism processes, and related functional categories (Figure 6C).

In the C2 group, DEGs showed enrichment in cellular and metabolic processes, macromolecular complexes, antioxidant activity, and related functional categories relative to those in the NDV group (Figure 6D), while comparison with the Test group revealed enrichment in the immune system, biological adhesion, growth, detoxification, regulation of molecular functions, and related functional categories (Figure 6E). Finally, DEGs in the NDV group were mainly enriched in cellular processes, growth, immune system, signal transducer activity, transcription factor activity, antioxidant activity, regulation of function factors, and related functional categories when compared with those in the Test group (Figure 6F).

By screening the GO functional annotations associated with the compound microecological preparation and NDV infection, we found that supplementation with the compound microecological preparation modulated the expression of genes involved in metabolic processes, biological regulation, immune response, growth and development, and related pathways. Combined with the clinical trial results, these findings demonstrated that the compound microecological preparation promoted intestinal development and enhanced intestinal immunity in chickens. Compared with microecological preparation supplementation, infection with type VII NDV induced changes in the expression of genes associated with the immune system, growth and development, functional factors, and related pathways. This indicated that NDV infection exerted a regulatory effect on intestinal development and immune function in chickens.

**Figure 7:**
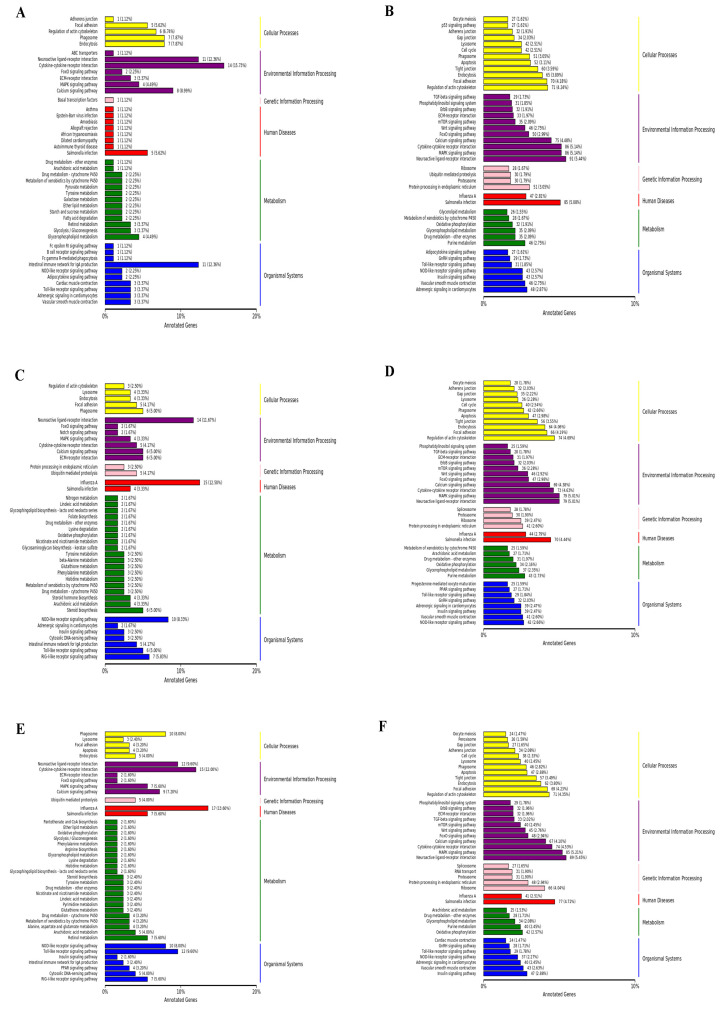
KEGG pathway enrichment analysis of differentially expressed genes. The ordinate represents the names of KEGG metabolic pathways, and the abscissa indicates the number of genes annotated to each pathway and their proportion relative to the total number of annotated genes. (**A**) C1_vs_C2; (**B**) NDV_vs_Test; (**C**) C1_vs_NDV; (**D**) C2_vs_NDV; (**E**) C1_vs_Test; (**F**) C2_vs_Test.

**Figure 8:**
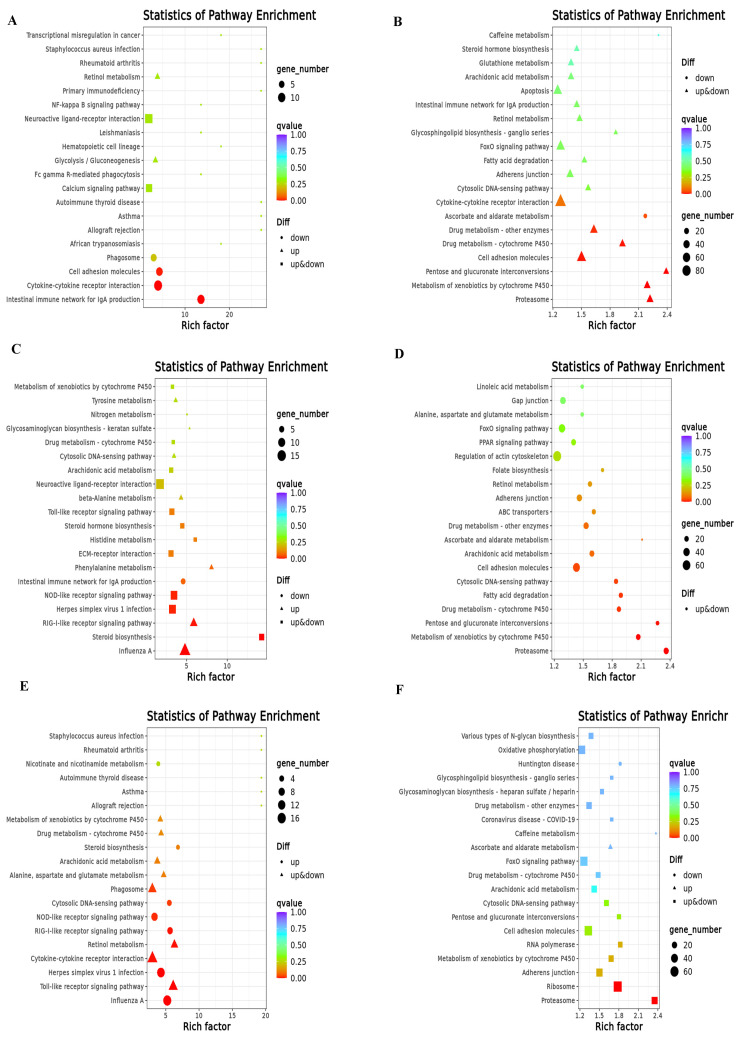
KEGG pathway enrichment distribution of differentially expressed genes. Each circle represents a KEGG pathway, the ordinate represents the name of the pathway, and the abscissa represents the enrichment factor. The larger the enrichment factor is, the more significant the enrichment level of differentially expressed genes in the pathway. Circle color represents the *q*-value, corresponding to the *p*-value adjusted for multiple hypothesis testing. A smaller *q*-value indicates greater reliability of the enrichment of differentially expressed genes in a given pathway. Circle size represents the number of genes enriched in a pathway; a larger circle represents more genes. (**A**) C1_vs_C2; (**B**) NDV_vs_Test; (**C**) C1_vs_NDV; (**D**) C2_vs_NDV; (**E**) C1_vs_Test; (**F**) C2_vs_Test.

Kyoto Encyclopedia of Genes and Genomes (KEGG) pathway enrichment analysis was performed, and the top significantly enriched signaling pathways are shown in Figure 7. Comparing C1 with C2, DEGs were predominantly enriched in immune-related pathways, including the intestinal immune network for IgA production, Toll-like receptor signaling, cytokine–cytokine receptor interactions, and bacteriophage-associated pathways. Notably, genes associated with the intestinal immune IgA pathway were highly enriched and showed a downregulation trend (Figure 8).

Compared with those in the NDV group, C1 DEGs were enriched in the NOD-like receptor signaling pathway, Toll-like receptor signaling pathway, and cytokine-related pathways, while comparison with the Test group further highlighted enrichment in the NOD-like receptor, RIG-I-like receptor, and Toll-like receptor signaling pathways.

In the C2 group, DEGs showed enrichment in the PPAR signaling pathway, NOD-like receptor signaling pathway, RIG-I-like receptor signaling pathway, and metabolic pathways when compared with the NDV group, whereas comparison with the Test group emphasized enrichment in the NOD-like receptor signaling pathway, Toll-like receptor signaling pathway, intestinal immune network for IgA production, and RIG-I-like receptor signaling pathway. Finally, compared with those in the Test group, DEGs in the NDV group were enriched in the MAPK, NOD-like receptor, and Toll-like receptor signaling pathways, drug metabolism, and related pathways. Combined with the GO functional annotation analysis, these results further demonstrated that the compound microecological preparation promotes the development of the intestinal immune system and enhances the immune response following NDV infection.

### 2.5. Screening of DEGs Related to Mucosal Immunity

Pairwise comparisons were conducted among the experimental groups, and 73 DEGs associated with mucosal immunity were screened from the data (Table 4). Among them, 11 DEGs were identified between the compound microecological preparation and control groups, 22 DEGs were screened between the virus-infected and control groups, and 15 DEGs were found between the compound microecological preparation and control groups under similar virus infection conditions.

## 3. Discussion

The mucosal immune system is an important component of the overall immune network in poultry. It possesses unique structural and functional characteristics and affects the ability of chickens to resist pathogenic infection. The intestinal mucosa of poultry is not only a key site for food digestion and nutrient absorption, but it is also the immune organ with the largest surface area and the highest number of immune cells, forming an effective defense system. In addition, the intestinal mucosa actively participates in antiviral defense and prevents pathogenic microorganisms from invading the gastrointestinal tract [6]. Physical barriers and mucosal immunity constitute the first line of defense against microbial infection. Therefore, enhancing intestinal mucosal immunity can improve overall immune function, strengthen resistance to pathogenic invasion, and prevent intestinal injury [7].

IgA-associated adaptive immunity plays a vital role in maintaining intestinal homeostasis. IgA-producing B cells reside in the lamina propria and secrete specific IgA, which is transported across the epithelium and secreted into the intestinal lumen. IgA regulates bacterial colonization, facilitates antigen presentation, and neutralizes pathogens to protect the host from intestinal infections. IgA can also trap bacteria in the mucus layer, promote phagocytosis, and kill bacteria that invade the mucosa [8]. Gut microbiota act as an antigen to stimulate IgA production, and their metabolite short-chain fatty acids (SCFAs) also influence IgA synthesis and the differentiation of B cells into plasma cells. sIgA is an important component of mucosal immunity, forming a protective barrier by adhering to epithelial cells or interfering with viral particle assembly by binding to newly synthesized viral proteins in infected cells. The protective potential of IgA is attributed to its polymeric properties, which result in high affinity for viruses and prevent viral adhesion to epithelial cells. The mucosal surface also serves as the first line of defense against infection through direct contact with foreign antigens [9,10,11]. Compound microecological preparations containing *Bacillus subtilis* and *Enterococcus faecium* modulate gut microbiota composition by selectively enriching these beneficial strains and suppressing opportunistic pathogen colonization. Their fermentation metabolites, particularly short-chain fatty acids (SCFAs), nourish intestinal epithelial cells to maintain barrier integrity, while also stimulating B cell differentiation and secretory IgA production to enhance mucosal defense against enteric pathogens.

Over the past few decades, antibiotics have been widely used as feed additives in poultry production to improve growth performance and intestinal health. However, since 1 July 2020, feed manufacturers have ceased production of commercial feed containing growth-promoting pharmaceutical additives (excluding traditional Chinese medicine products), in accordance with Announcement No. 194 of the Ministry of Agriculture and Rural Affairs of the People’s Republic of China. Following the ban on antibiotic use in poultry feed, poultry increasingly rely on their own immune systems to defend against various threats during growth. New functional additives are urgently needed to improve production performance, enhance immunity, prevent pathogen invasion, and promote the sustainable development of animal husbandry. To this end, researchers and feed producers are exploring safer alternatives.

Various microorganisms have been widely used as probiotics in poultry production, including *Bacillus*, *Lactococcus*, and yeast, among others. For example, Xu et al. [12] investigated the effects of dietary supplementation with *Bacillus subtilis* or *Bacillus licheniformis* on growth performance, immunity, antioxidant capacity, SCFA production, and cecal microflora in broilers. Their results showed that both *B. subtilis* and *B. licheniformis* significantly increased body weight and average daily gain, markedly elevated serum levels of IgA, IgY, IgM, and the anti-inflammatory cytokine IL-10, and significantly decreased the pro-inflammatory cytokines IL-1β and IL-6. GO and KEGG enrichment analyses revealed that the compound microecological preparation containing these two *Bacillus* strains induced changes in the expression of genes involved in signaling pathways related to growth and development, antioxidant activity, and intestinal immunity (Figure 6 and Figure 8), consistent with the intestinal morphological changes observed in clinical necropsy (Figure 2). Helmy et al. [13] reported that probiotic supplementation with *Escherichia coli* Nissle 1919 reduced *Campylobacter jejuni* colonization in chickens and increased jejunal and ileal villus height, thus controlling *Campylobacter* infection in poultry. Muthusamy et al. [14] found that dietary supplementation with yeast cell wall product significantly increased the level of specific antibodies against NDV in broilers. Collectively, these studies indicate that certain probiotics can indeed enhance immunity and exert antiviral effects.

Huang et al. [6] found that dietary litchi (*Litchi chinensis*) pulp polysaccharides improved intestinal mucosal immune function by stimulating mesenteric lymph node cell proliferation and serum IgA secretion. Wu et al. [15] demonstrated that treatment with *Hericium erinaceus* polysaccharides significantly improved intestinal mucosal morphology in partridge ducks, increased the number of immune cells and the production of secretory IgA (sIgA) and cytokines in the intestinal mucosal immune system, and reversed damage to the intestinal mucosal immune barrier. Zhou et al. [16] extracted polysaccharides from *Pinus massoniana* pollen collected from Mount Taishan and investigated their intestinal mucosal immunity–promoting effects. The results showed that Taishan pine pollen polysaccharides significantly increased serum antibody and mucosal secretory IgA levels, intestinal villus integrity, and the expression of cytokine genes and mucosal immunity–related genes in chickens. Specifically, it reduced intestinal pathological injury and viral load following NDV vaccination, further confirming that Taishan pine pollen polysaccharides enhance mucosal immunity and promote intestinal villus development. Deng et al. [17] found that Yupingfeng polysaccharides significantly enhanced the activation of inductive sites in the intestinal mucosa, stimulated sIgA secretion, and regulated both local and systemic immune responses. *Astragalus* polysaccharides (APSs) are the principal components of water-soluble heteropolysaccharides extracted from the stems and dried roots of *Astragalus membranaceus*, a traditional Chinese medicinal herb. APSs possess multiple biological activities, including antioxidant, anti-inflammatory, antibacterial, and antiviral properties, along with immunomodulatory functions [18]. This is consistent with the results of the present study, where the APS-containing microecological preparation triggered alterations in signaling pathways related to intestinal immunity and antioxidant activity (Figure 7).

Li et al. [19] found that APSs inhibit adverse morphological changes in the intestinal mucosa, while increasing the number of immunocompetent cells in the jejunal mucosa and upregulating the mRNA expression of several intestinal cytokines. Wang et al. [20] investigated the effects of xylo-oligosaccharides and APSs on immune response, antioxidant capacity, and intestinal microbiota composition in broilers. They found that their combined use improved intestinal mucosal immunity and barrier function in broilers by upregulating cytokine gene expression, increasing the number of IgA-producing cells, and modulating cecal microbiota. GO and KEGG enrichment analyses in the present study revealed that the compound microecological preparation significantly upregulated cytokine gene expression and promoted intestinal IgA production (Figure 7 and Figure 8). Qiao et al. [21] investigated the effects of dietary supplementation with APSs and *Glycyrrhiza* polysaccharides on growth performance, intestinal health, and intestinal microbiota composition in broilers. Their results demonstrated that APSs and *Glycyrrhiza* polysaccharides increased body weight gain, reduced the feed-to-gain ratio, and improved intestinal health in broilers by ameliorating intestinal morphology and mucosal barrier function. Consistent with these findings, our study revealed that the APS-containing compound microecological preparation delayed disease progression and promoted intestinal growth. As shown in Figure 2, the preparation exerted a protective effect on the cecal tonsil mucosa of chickens following NDV infection.

## 4. Materials and Methods

### 4.1. Viruses, Experimental Animals, and Other Materials

Genotype VII NDV strain (GN-17) and 9–11-day-old specific pathogen-free (SPF) chicken embryos were purchased from Shaanxi Yangling Lvfang Bioengineering Co., Ltd. (Xianyang, China). The embryos were incubated in a controlled incubator and reared in animal isolators. The compound microecological preparation was prepared in our laboratory using *B. subtilis*, *B. licheniformis*, *Enterococcus faecalis*, *Clostridium butyricum*, lactic acid bacteria, and APSs. The Reverse Transcription Kit and ready-to-use PCR Premix were obtained from Beijing TransGen Biotech Co., Ltd. (Beijing, China); the DNA Marker and Real-Time PCR Kit were purchased from GenStar (Beijing, China); and RNAiso Plus was obtained from Takara Bio Inc. (Shiga, Japan).

### 4.2. Experimental Design and Sample Collection

Clinically healthy 4-week-old specific pathogen-free (SPF) chickens were randomly allocated to 4 experimental groups, with 10 chickens per group, using a computer-generated random number sequence. The randomisation process was performed by an independent researcher not involved in subsequent animal handling, viral challenge and data collection to minimise selection bias. In the event of accidental death unrelated to viral challenge, severe trauma, or failure to receive the designated treatment/inoculation, such animals would have been excluded from all subsequent analyses. The C1 and NDV groups were fed a standard complete feed (Shaanxi Huaqin Agrictech Co., Ltd., Yangling, Shaanxi, China.), while the C2 and Test groups received the same diet supplemented with the compound microecological preparation at a ratio of 1000:1 (i.e., 1 g of the preparation added to 1000 g of feed). Other feeding conditions were consistent across all groups. After 2 weeks of feeding, the NDV and Test groups were subjected to an NDV challenge test. GN-17 virus allantoic fluid with an HI titer of 2^8^ was inoculated at 150 μL per chicken via ocular–nasal instillation, while the C1 and C2 groups were treated with an equal volume of PBS solution via the same route. Five chickens in each group were used for observation of clinical signs and pathological changes. After necropsy of the other five chickens, a 1 cm intestinal segment from each side of the cecal tonsil was collected for transcriptome sequencing. For transcriptome sequencing, standard industry quality control (QC) procedures were followed a priori: raw reads with Q30 < 80% or genome mapping rate < 70% would have been excluded. All 20 transcriptome samples in this study met the QC requirements, and no data were excluded. Sequencing services were provided by Beijing Tsingke Biotech Co., Ltd. (Beijing, China).

To minimise confounding, cage locations were randomised via computer-generated sequence prior to the experiment. All treatments, NDV challenge, daily observations and sample collections were performed in random order across groups, with observation sequences regenerated daily. All chickens were housed under identical environmental conditions with ad libitum feed and water, and all procedures were conducted by a single trained researcher to eliminate inter-operator variability.

### 4.3. Intestinal Transcriptome Analysis

Total RNA was extracted using Trizol reagent (Tiangen Biotech, Beijing, China) following the manufacturer’s instructions. The concentration of the extracted nucleic acids was quantified using a Nanodrop 2000 spectrophotometer (Thermo Fisher Scientific, Waltham, MA, USA), and their integrity was assessed using an Agilent 2100 Bioanalyzer (Agilent Technologies, Santa Clara, CA, USA) and LabChip GX system (Revvity Inc., Hopkinton, MA, USA). mRNA Capture Beads were thoroughly mixed on a four-dimensional rotator and equilibrated for 30 min prior to use. For mRNA purification and fragmentation, the beads were added to the prepared total RNA samples, mixed gently, and incubated at 65 °C for RNA denaturation, followed by incubation at room temperature for 5 min to allow mRNA binding to the magnetic beads. The samples were placed on a magnetic stand until complete bead separation, and the supernatant was discarded. The beads were washed twice with Wash Buffer, resuspended in Tris Buffer, and mRNA was eluted at 80 °C. Beads Binding Buffer was then added, and the mixture was incubated at room temperature for 5 min to allow mRNA rebinding to the beads. After magnetic separation, the supernatant was removed, the beads were washed once with Wash Buffer, and Frag/Prime Buffer was added. The mRNA mixture was transferred to a PCR instrument, and fragmentation conditions were determined based on sample quality. Immediately after fragmentation, the samples were placed on ice. First-strand and second-strand cDNA were synthesized sequentially, followed by product purification, end repair, and 3′-end A-tailing. Adapters were ligated to the cDNA fragments using T4 DNA ligase with incubation in a metal bath. After purification of the ligation products and size selection, PCR reaction reagents were added, mixed thoroughly, and centrifuged briefly. PCR amplification was performed according to the optimized reaction conditions, followed by purification of the PCR products. The final cDNA libraries were aliquoted and stored for subsequent sequencing.

### 4.4. Data Processing and Analysis

After sequencing was completed, the raw data were processed using CASAVA base calling, and the resulting output was converted into raw sequencing reads (raw data), which were saved in FASTQ (abbreviated as fq) format. The raw data were filtered to obtain clean reads for sequence alignment. Based on the alignment results, analyses, including novel transcript prediction, as well as single-nucleotide polymorphism and Indel detection, were performed. Quantitative analysis was conducted on known genes and the identified novel genes to investigate gene expression patterns across different sample groups, followed by differential expression analysis. For the identification of DEGs, the screening criteria were set as Log_2_ Fold Change ≥ 1 and *p*-value < 0.05. The DEGs screened using these criteria were subjected to GO functional, KEGG pathway, and cluster analyses.

### 4.5. qPCR

Viral RNA was extracted using RNAiso Plus (Takara Bio Inc., Shiga, Japan). After determination of RNA concentration, RNA was uniformly diluted to 100 ng µL^−1^, and cDNA was synthesized using the StarScript First-strand cDNA Synthesis Mix kit (GenStar, Beijing, China). The reverse transcription program was set as follows: 25 °C for 10 min, 42 °C for 30 min, and 85 °C for 5 min. The synthesized cDNA was stored at −20 °C for subsequent use. qPCR was conducted using a 2× RealStar Power dye-based qPCR kit (GenStar), according to the manufacturer’s instructions, with 28S rRNA as the internal reference gene to quantify target gene expression. The qPCR reaction system was prepared on ice (Table 5). The thermal cycling condition included pre-denaturation at 95 °C for 10 min, followed by 40 cycles of 95 °C for 15 s, 60 °C for 1 min, and 72 °C for 30 s.

## 5. Conclusions

This study investigated the pathogenic effects of a compound microecological preparation on chickens infected with genotype VII Newcastle disease virus (NDV, GN-17) using clinical trials and transcriptome sequencing technology. It was found that the compound microecological preparation could significantly enhance immune efficacy, serve as an immunopotentiator for clinical production, promote intestinal development in chickens, delay disease progression, and reduce infection-induced damage. Additionally, the compound microecological preparation could promote the production of IgA in the intestinal immune system and induce changes in the expression of multiple genes involved in intestinal immune system-related pathways, such as the NOD-like receptor signaling pathway, RIG-I-like receptor signaling pathway, Toll-like receptor signaling pathway, and PPAR signaling pathway. These findings provide a scientific basis and research foundation for the effective prevention and control of Newcastle disease. Subsequent studies will focus on screening core differential genes in the above-mentioned key immune and signaling pathways, conducting targeted in-depth mechanism verification research, and clarifying the specific regulatory role and interactive molecular mechanism of core genes in the process of the compound microecological preparation regulating intestinal immunity and resisting Newcastle disease virus infection. This follow-up research can accurately clarify the core molecular targets of the preparation’s antiviral and immunomodulatory effects, improve the molecular theoretical system of the compound microecological preparation for preventing and controlling avian Newcastle disease, and lay a solid core scientific research foundation for the precise optimization and improvement of the preparation and its large-scale clinical application.

## Figures and Tables

**Table 1:** Statistics of differentially expressed genes.

DEG Set	DEG Number	Upregulated	Downregulated
C1-1_C1-2_C1-3_vs_C2-1_C2-2_C2-3	329	104	225
C1-1_C1-2_C1-3_vs_NDV-1_NDV-2_NDV-3	5276	2673	2603
C1-1_C1-2_C1-3_vs_Test-1_Test-2_Test-3	346	214	132
C2-1_C2-2_C2-3_vs_NDV-1_NDV-2_NDV-3	5017	2588	2429
C2-1_C2-2_C2-3_vs_Test-1_Test-2_Test-3	444	305	139
NDV-1_NDV-2_NDV-3_vs_Test-1_Test-2_Test-3	5080	2488	2592

DEG, differentially expressed gene.

**Table 2:** Primers used in this study.

Primers	Sequences (5′–3′)	Method	Size	References
IFI35	F: AGGGAGTTCCTGGATGAC	qPCR	229 bp	Present study
	R: GCTCCTCAGCCAGCACAT			
CHN2	F: TACAGAGTCTCAGGCTTCAC	qPCR	195 bp	Present study
	R: TGCTGCCTCTATGAACTTGG			
CTSB	F: GCCTCAGCTAGAAGAGAAAG	qPCR	192 bp	Present study
	R: CTGCTCTGGTATCCATGAAG			
OASL	F: AGATGTTGAAGCCGAAGTACCC	qPCR	106 bp	[5] DOI: 10.1007/s11262-014-1151-z
	R: CTGAAGTCCTCCCTGCCTGT			
CD82	F: GGAGTTGTCACGCATAGTTG	qPCR	181 bp	Present study
	R: GGGATACGCAGTCATGCTTC			
TLR2A	F: TCACCATGAGGCAGAGATAG	qPCR	183 bp	Present study
	R: ACCAGGATGAGGATGAACAG			
CD40	F: TGGCCTCATTGTGAAGAGAC	qPCR	202 bp	Present study
	R: ATTGGAGAAGGTGCCTTCTG			
IRF7	F: CAGTGCTTCTCCAGCACAAA	qPCR	169 bp	Present study
	R: TGCATGTGGTATTGCTCGAT			
IFIH1	F: TTGTCAGAGAGAGCAGTGTATTGGA	qPCR	109 bp	Present study
	R: GAATCACTGGTCGTGCTGCTCTGTC			
DHX58	F: CCAGAATGAGCAGCAGGAC	qPCR	109 bp	Present study
	R: AATGTTGCACTCAGGGATGT			
28S	F: GGTATGGGCCCGACGCT	qPCR	160 bp	Present study
	R: CCGATGCCGACGCTCAT			

**Table 3:** Statistics of annotated differentially expressed genes.

DEG Set	Total	Different Function Database							
		COG	GO	KEGG	KOG	NR	Pfam	Swiss-Prot	Egg NOG
C1-1_C1-2_C1-3_vs_C2-1_C2-2_C2-3	313	75	191	182	133	310	267	274	293
C1-1_C1-2_C1-3_vs_NDV-1_NDV-2_NDV-3	5103	1600	3591	3503	3379	5056	4618	4808	4946
C1-1_C1-2_C1-3_vs_Test-1_Test-2_Test-3	326	104	207	203	178	320	282	291	313
C2-1_C2-2_C2-3_vs_NDV-1_NDV-2_NDV-3	4854	1549	3439	3337	3261	4809	4397	4577	4712
C2-1_C2-2_C2-3_vs_Test-1_Test-2_Test-3	419	134	264	256	201	411	363	374	400
NDV-1_NDV-2_NDV-3_vs_Test-1_Test-2_Test-3	4926	1565	3474	3385	3300	4882	4455	4633	4777

DEG, differentially expressed gene; COG, Clusters of Orthologous Groups; GO, Gene Ontology; KEGG, Kyoto Encyclopedia of Genes and Genomes; KOG, Eukaryotic Orthologous Groups; NR, NCBI non-redundant protein database; Pfam, Protein family database; Swiss-Prot, manually curated protein sequence database; Egg NOG, Evolutionary Genealogy of Genes: Non-supervised Orthologous Groups.

**Table 4:** Screening of genes associated with the intestinal immune network for IgA production.

Groups	ko ID	DEG Number	DEG Name
C1_vs_C2	ko04672	11	*MSTRG.23708*; *MSTRG.23710*; *gene-AICDA*; *gene-CCR10*; *gene-CD28*; *gene-CXCR4*; *gene-ICOS*; *gene-ITGA4*; *gene-LOC107049666*; *gene-LOC107051274*; *gene-MADCAM1*
C1_vs_NDV	ko04672	22	*MSTRG.15393*; *MSTRG.1765*; *MSTRG.23708*; *MSTRG.23712*; *gene-AICDA*; *gene-BLB2*; *gene-CCR10*; *gene-CCR9*; *gene-CD28*; *gene-CD40LG*; *gene-CD80*; *gene-CXCL12*; *gene-DMA*; *gene-ICOS*; *gene-ICOSLG*; *gene-IL15RA*; *gene-IL6*; *gene-LOC107049666*; *gene-LOC107051274*; *gene-MADCAM1*; *gene-PIGR*; *gene-TNFSF13B*
C1_vs_Test	ko04672	5	*MSTRG.15393*; *MSTRG.1765*; *gene-AICDA*; *gene-CD28*; *gene-LOC107049666*
C2_vs_NDV	ko04672	17	*MSTRG.15393*; *MSTRG.23708*; *gene-CCR9*; *gene-CD40*; *gene-CD40LG*; *gene-CD80*; *gene-CXCR4*; *gene-DMA*; *gene-ICOS*; *gene-ICOSLG*; *gene-IL15RA*; *gene-IL6*; *gene-LOC107049666*; *gene-LOC107051274*; *gene-MADCAM1*; *gene-MAP3K14*; *gene-PIGR*
C2_vs_Test	ko04672	3	*MSTRG.23708*; *MSTRG.23710*; *gene-MADCAM1*
NDV_vs_Test	ko04672	15	*MSTRG.23708*; *MSTRG.23710*; *MSTRG.23712*; *gene-AICDA*; *gene-CD28*; *gene-CD40LG*; *gene-CD80*; *gene-CD86*; *gene-DMA*; *gene-ICOS*; *gene-ICOSLG*; *gene-IL6*; *gene-MADCAM1*; *gene-PIGR*; *gene-TNFSF13B*

DEG, differentially expressed gene.

**Table 5:** qPCR reaction system used in this study.

Components	Volume (μL)
cDNA	1.0
Forward primer (10 μM)	0.5
Reverse primer (10 μM)	0.5
2× RealStar Power SYBR qPCR Mix	10.0
ddH_2_O	8.0

## Data Availability

We confirm that the full raw sequencing data are available to support all analyses in the manuscript. The data can be deposited in a public database with an official accession ID provided upon request (estimated turnaround time: ~4 weeks). The raw data are also available from the corresponding author on request.

## Supplementary Materials

The following supporting information can be downloaded at: https://www.mdpi.com/article/10.3390/ijms27135771/s1.

## Abbreviations

The following abbreviations are used in this manuscript:

APS	*Astragalus* polysaccharides
DEG	Differentially expressed genes
GO	Gene Ontology
KEGG	Kyoto Encyclopedia of Genes and Genomes
ND	Newcastle disease
NDV	Newcastle disease virus
SCFA	Short-chain fatty acids
SPF	Specific pathogen-free
