# Supplementary material for: Transcriptome Analysis of the Effects of Compound Microecological Preparation on Chickens Challenged with Newcastle Disease Virus
Source: Int J Mol Sci. 2026 Jun 26;27(13):5771. doi: 10.3390/ijms27135771 (PMC13360924; doi:10.3390/ijms27135771)
Supplement: Supplementary file 1 [file ijms-27-05771-s001.zip › ijms-4355982-supplementary.pdf]

Supplementary Materials:

We have completed the clinical sign and pathological lesion scoring records for all ten chickens per group in strict accordance with the standardized scoring criteria established in our previous study. The detailed scoring criteria are referenced from [5].

Table S1 Scoring record of clinical signs and pathological lesions

Types / Groups	Clinical sign scoring										Pathological lesion scoring									
	1-3 dpi					4-7 dpi					1-3 dpi					4-7 dpi				
	1	2	3	4	5	6	7	8	9	10	1	2	3	4	5	6	7	8	9	10
C1	0	0	0	0	0	0	0	0	0	0	0	0	0	0	0	0	0	0	0	0
NDV	4	3	4	3	3	4	4	4	4	4	3	3	3	2	3	4	3	2	3	3
C2	0	0	0	0	0	0	0	0	0	0	0	0	0	0	0	0	0	0	0	0
Test	1	1	2	1	1	2	4	4	4	4	1	0	1	1	1	1	2	2	2	3
